# How gratitude and admiration differently enhance sustainable consumer behavior

**DOI:** 10.3389/fpsyg.2025.1584263

**Published:** 2025-07-16

**Authors:** Cheng Che, Miaomiao Zhou, Suhui Li

**Affiliations:** School of Economics and Management, China University of Petroleum (East China), Qingdao, China

**Keywords:** emotions, gratitude, admiration, human values, sustainable behavior

## Abstract

Prior research indicates that various emotions linked to distinct values can result in unique consumer behaviors. The research is based on the discrete emotion theory, human value theory, and the theory of emotional-value consistency. It uses the experimental method to explore the influence of two completely different positive emotions, gratitude and admiration, on consumers’ willingness to engage in sustainable behaviors. It is based on the concepts of differentiated emotions, human values, and the congruence between emotions and values. The research demonstrates that personal values influence the effectiveness of demonstrations of gratitude and admiration. When self-transcendence values are prioritized, gratitude increases customers’ likelihood of engaging in sustainable behaviors; conversely, when self-enhancement values are emphasized, admiration heightens consumers’ motivation to act sustainably. Self-efficacy clarifies this concept. Two investigations validated these impacts, augmenting our comprehension of the factors influencing sustainable consumer behavior, which benefits both marketers and regulators.

## 1 Introduction

All sectors of society have acknowledged sustainable practices with the establishment of the “double carbon” aim. An increasing number of enterprises are promoting sustainable practices through many techniques, including reducing greenhouse gas emissions, supporting environmental organizations, utilizing recyclable packaging, and manufacturing products with eco-friendly materials. The Thanksgiving Day tagline of Golden Dawn Organic Milk exemplifies how numerous companies leverage customer emotions in their marketing to promote sustainable consumption. We can continue to advance due to your passion and your rigorous, analytical reasoning. Our ability to remain united is attributable to your presence.

Through an analysis of existing literature, we discovered that the impact of the two emotions, gratitude and admiration, on sustainable behavior remains ambiguous. Regarding the impact of gratitude on sustainable behavior, while certain studies indicate that gratitude can enhance individuals’ pro-environmental intentions, diminish resource-wasting actions, and elevate sustainable product selections (Liang et al., 2019; Septianto et al., 2020; Syropoulos et al., 2020; Liang and Guo, 2021), further pertinent research is required to elucidate the mechanisms and timing through which gratitude fosters sustainable behaviors. Nonetheless, there is no direct evidence demonstrating the impact of admiration on sustainable behaviors; admiration has only been shown to affect pro-social activities through indirect evidence (Algoe and Haidt, 2009; Vianello et al., 2010). Research indicates that self-transcendent emotions, such as gratitude and admiration, might convey pro-social tendencies to others (Stellar et al., 2017). Sustainable behavior differs subtly from pro-social behavior, with the latter encompassing a wide range of actions deemed advantageous to others by social groupings (Penner et al., 2005). Sustainable behavior considers the wellbeing of both humanity and the environment. Consequently, additional study is required to validate the influence of admiration on sustainable behavior. In conclusion, while gratitude and admiration are favorable feelings frequently employed by marketers, the academic community lacks a comprehensive knowledge of their effects and underlying psychological mechanisms (Onu et al., 2016; Jacobs and McConnell, 2024). A comprehensive study of the distinct effects of gratitude and admiration could assist marketers and policymakers in formulating more precise and effective campaigns to encourage sustainable behaviors. This paper seeks to examine the distinct benefits of gratitude and admiration on sustainable behaviors, beyond their general positive influences.

The evaluative tendency framework posits that emotions correlate with particular evaluations, and once activated, these emotions instigate a cognitive predisposition to assess subsequent events according to the fundamental evaluative dimensions that elicited the emotion (certainty, sense of control), thereby affecting decision-making behavior (Han et al., 2007). Consequently, these varied evaluative dimensions differentiate among distinct emotions and thus influence how individuals finally resolve issues (Ellsworth and Scherer, 2002; Keltner and Horberg, 2015). From this viewpoint, while gratitude and admiration are both positively experienced emotions, their fundamental evaluative characteristics differ. Gratitude primarily emphasizes appreciation for others and fosters a rewarding mentality (Wood et al., 2008), whereas admiration represents a shift in emphasis that promotes an enhanced perspective upon recognizing the greatness of others (Chen, 2019). The disparity in evaluative dimensions may result in varying impacts of gratitude and admiration on individuals’ conduct (Piff et al., 2015). Research indicates that various emotions affect individuals’ product assessment and purchasing behavior (Kranzbühler et al., 2020). Consequently, examining the impact of various emotions on sustainable behaviors might aid in the formulation and enhancement of marketing tactics.

It is important to acknowledge that while emotions affect consumer decision-making, they are simultaneously shaped by individual values at the time of the decision (Goenka and Van Osselaer, 2019). Human values influence the trajectory of individual behavior (Schwartz, 1992), and brands can embody these human values (Torelli et al., 2012). In reality, numerous brands are imbued with distinct values by marketers (Rodas et al., 2021). For instance, Louis Vuitton, as a premier fashion brand, often embodies the aspiration for a luxurious lifestyle, communicates the yearning for personal influence and social standing, and illustrates the pursuit of self-enhancing values. Conversely, Philips, as a health technology brand, reflects its values through a commitment to social responsibility, sustainable development, innovation, and business integrity, thereby aligning itself with the principle of self-transcendence. The priority of values influences individuals’ understanding of emotional experiences (Tamir et al., 2016), while the fundamental evaluative characteristics of emotions further impact decision-making. Research indicates that self-transcendent emotions, such as awe, compassion, and love, correlate with more sustainable behaviors, attitudes, values, and self-representations, as these emotions foster a pro-social mindset and expand the self-concept (Jacobs and McConnell, 2024). We claim that the interplay between discrete emotions and values might substantially affect consumers’ decisions regarding sustainable behavior.

Research indicates that various emotions can be differentiated according to distinct values (Stellar et al., 2017), and that emotions emphasize the identification of an individual’s sensitivity to specific values (Haidt, 2003). Given that gratitude is linked to self-transcendent value evaluations (Wood et al., 2008) and admiration is connected to self-enhancing value evaluations (Chen, 2019), subsequent sections will elucidate how the congruence of specific positive emotions with human values bolsters consumers’ self-efficacy (Locke and Bandura, 1987) and consequently promotes sustainable behavior. This paper will examine two research questions:

1. Whether the interplay of the positive discrete emotions of gratitude and admiration with human values fosters sustainable behavior.
2. The mechanisms through which these emotions facilitate sustainable behavior. The study will address the deficiency of research about the impact of gratitude and admiration on sustainable behavior and will further enhance the pertinent literature. The study will assist marketers in emphasizing the utilization of gratitude and admiration—two affirmative emotions—in advertising and brand publicity to encourage sustainable consumer behavior, thereby aiding in the achievement of the dual-carbon objective and the pursuit of sustainable development.

## 2 Theoretical framework and hypothesis projection

### 2.1 Sustainable behavior and distinct emotions

Sustainable behaviors are actions that preserve resources and mitigate adverse environmental effects throughout the life cycle of a product, behavior, or service. This includes selecting eco-friendly products, conserving resources during usage, and disposing of recyclables sustainably (White et al., 2019). Sustainable activities advantage a broader spectrum of individuals and the environment, in contrast to pro-social actions, which solely benefit others (Nolan and Schultz, 2013). Consequently, same emotions may have varying impacts on pro-social behavior and sustainable behavior, necessitating further investigation into their influence on sustainable behavior.

Presently, both local and international researchers have undertaken numerous studies on the influence of emotions on consumer behavior, encompassing its impact on sustainable practices. Initial research predominantly emphasized the influence of negative emotions on sustainable behavior, including shame and sadness (Rees et al., 2015; Schwartz and Loewenstein, 2017). It is only in recent years that academics have progressively shifted their focus to the impact of positive emotions, such as pride, gratitude, and affection (Septianto et al., 2021; Wang et al., 2017). Research indicates that positive emotions, particularly self-transcendent feelings like speculate and gratitude, appear to encourage pro-environmental activities (Zelenski and Desrochers, 2021). It is crucial to highlight that various positive emotions have distinct functions in augmenting persuasion (Karsh and Eyal, 2015), and the resultant impacts on specific behaviors varies (Schneider et al., 2017). Scholars are increasingly recognizing that each pleasant emotion possesses distinct triggers, behavioral inclinations, cognitions, and effects, warranting discrete study (Piff et al., 2015; Prade and Saroglou, 2016; So et al., 2015). This paper examines the impact of two positive emotions, gratitude and admiration, on consumers’ propensity to engage in sustainable behavior.

### 2.2 Gratitude

Gratitude is a pleasant feeling characterized by an individual’s appreciation and desire to repay upon recognizing and experiencing altruistic assistance from external sources. The positive expansion construct theory of gratitude posits that gratitude aids individuals in sustaining happy feelings, mitigates the detrimental impacts of negative emotions, and enables adaptation to their settings while fostering a positive outlook on life (Fredrickson, 2004). Initial scholars primarily examined gratitude as a moral feeling; however, the emergence of psychology has prompted increased focus on gratitude as a pleasant emotion. Academics currently classify gratitude into two primary forms: state gratitude and trait gratitude (McCullough et al., 2002). State gratitude refers to an immediate experience of gratitude linked to a particular circumstance. Trait gratitude is a persistent inclination to feel appreciation, characterized by its stability and independence from contextual factors (Wood et al., 2010). The trait of gratitude is linked not only to the intention to engage in pro-environmental behaviors but also to the act of donating to environmental charities (Tam, 2022). Recent studies indicate that the impact of gratitude is contingent upon several elements, including the recipient of thanks, the situational setting, and individual variances. Gratitude can augment an individual’s perception of social connectivity (Algoe, 2012) and foster pro-social activities (Ma et al., 2017), including volunteering, charitable contributions, and, to a degree, pro-environmental actions such as composing thank-you cards (Jacobs and McConnell, 2024). In consumer behavior, gratitude enhances brand loyalty and customer satisfaction, consequently affecting purchase decisions and word-of-mouth communication (Liang and Guo, 2021; McCullough et al., 2002; Dunn and Schweitzer, 2005; Emmons and McCullough, 2003). Moreover, gratitude is positively correlated with individuals’ wellbeing and life satisfaction, and it enhances psychological wellbeing (Emmons and McCullough, 2003; Seligman et al., 2005).

### 2.3 Admiration

Admiration emphasizes the qualities and strengths of the object, whereas gratitude centers on the appreciation of assistance provided by others (Algoe and Haidt, 2009). Admiration in positive psychology is characterized as the recognition and respect for the commendable traits or actions of others (Chen et al., 2011; Ortony et al., 2022). Admiration, in contrast to other positive emotions, activates individuals’ motives for imitation and belonging, thereby fostering self-improvement and pro-social behaviors (Immordino-Yang et al., 2009; Onu et al., 2016; Sarapin et al., 2015). Admiration is associated with an individual’s self-efficacy and outcome expectations, impacting career intentions and behaviors (Sarapin et al., 2015). Research indicates that admiration enhances altruistic and cooperative behaviors among individuals (Algoe and Haidt, 2009; Vianello et al., 2010). In the study of sustainable behavior, admiration has been shown to indirectly promote the adoption of sustainable practices; however, direct research in this area is insufficient, and additional empirical studies are necessary to further investigate the relationship between admiration and sustainable behavior (Cialdini et al., 1991). The impact of admiration on sustainable behavior is influenced by individual and cultural differences. Individual differences (Schwartz, 1992) and cultural context (Schneider et al., 2017) may influence the effect of admiration. Thus, analyzing the conditions under which admiration fosters sustainable behavior can enhance comprehension of the extent and limitations of admiration’s pro-social effects. This study offers practical insights and recommendations for marketers on effectively employing admiration appeals in relation to brand value and consumer value priorities.

### 2.4 Interaction of gratitude, admiration and human values

Several scholars have introduced the Appraisal Cognitive Tendency Framework (ATF), which is grounded in the cognitive appraisal theory of emotion and the functional theory of emotion (Lerner and Keltner, 2000, 2001). The cognitive appraisal theory of emotion posits that the ATF highlights the role of specific emotions in decision making, rather than their intensity, which is categorized as positive or negative (Smith and Ellsworth, 1985). Emotions consist of multidimensional cognitive appraisals, with core appraisal themes identified as the central dimensions in this process (Han et al., 2007). The six cognitive appraisal dimensions of emotion include certainty, pleasure, attention, control, maneuverability, and responsibility (Smith and Ellsworth, 1985). The functional theory of emotion posits that the ATF highlights the coordinating function of emotion, facilitating swift problem-solving by redirecting an individual’s cognitive attention to the event that elicited the emotion (Johnson-Laird and Oatley, 1992). Furthermore, an individual’s cognitive control over emotions may be sufficiently robust to elicit related cognitions concerning events distinct from the original event (Lerner and Keltner, 2000).

The ATF posits that the fundamental evaluative themes of an emotion trigger cognitive dispositions aligned with an individual’s basic cognitive evaluative dimensions, which inform their judgment of events and influence decision-making processes. The evaluative tendencies process involves the influence of emotion on an individual’s cognition, which subsequently modifies judgment and decision-making, ultimately addressing the problem that elicited the emotion (Lerner and Keltner, 2000). Different emotions are categorized based on core evaluative themes, which influence both the type of emotion experienced and the direction of the individual’s final decision. Future events must align with core evaluative themes to generate evaluative tendencies (Han et al., 2007). If an event elicits multiple emotions in an individual, but the primary attribute of this event is responsibility, then only emotions associated with responsibility will influence the individual’s cognitive appraisal of the event.

Utilizing a combination of evaluative cognitive disposition theory and Schwartz’s theory of human values, this study examines human values as a central evaluative theme to explore the impact of various emotions on sustainable behaviors in relation to different dimensions of values (Yan et al., 2024). Schwartz’s theory of human values serves to explain and categorize the values individuals possess, highlighting that these values can be understood along a continuum and may coexist within the same value domain (Schwartz, 1992). Schwartz’s human values model identifies 10 fundamental values, organized into four domains according to their association with abstract goals, specifically goal motivation: self-enhancement, self-transcendence, conservatism, and change (Schwartz et al., 2012). The Self-Enhancement (SE) and Self-Transcendence (ST) dimensions are employed in this paper to continue the previous research paradigm (Yan et al., 2024). The two dimensions illustrate the varying emphasis individuals place on interests, with one dimension prioritizing self-interest and the other emphasizing the wellbeing of others (Schwartz, 1992). Self-enhancement specifically highlights external power and influence, illustrating an individual’s motivation to pursue self-enhancement and augment personal benefits (Schwartz et al., 2012). It includes sub-values such as power, success, status, competence, and influence (Burson et al., 2012; Schwartz and Boehnke, 2004). Self-transcendence prioritizes the pursuit of transcendence beyond personal interests, highlighting a heightened concern for others and demonstrating individual selflessness in relation to the collective good (Schwartz et al., 2012). It includes sub-values such as responsibility, helpfulness, environmental protection, and caring (Burson et al., 2012; Schwartz and Boehnke, 2004). The present research examines the correlation between the emotions of gratitude and admiration and the values of self-transcendence and self-enhancement, influencing individuals’ perceptions of sustainable behaviors in decision-making processes. Gratitude is defined by helpfulness, indicating an individual’s appreciation and concern for others (Wood et al., 2008). Secondly, gratitude primarily highlights the motivational aspect of focusing on others’ interests and reflects self-transcendent values. Admiration pertains to the appreciation of the abilities and virtues of positive entities, which can activate an individual’s motives for imitation and belonging, thereby facilitating self-enhancement (Chen, 2019). Admiration is directed toward the balance between self-transcendence and self-interest, thereby illustrating self-enhancing values. This paper posits that the alignment of gratitude with self-transcendence values and admiration with self-enhancement values enhances the likelihood of engaging in desired sustainable behaviors. We propose the following hypothesis:

*H1:* An interaction exists between discrete positive emotions and human values, specifically:

- Gratitude enhances sustainable behavior when self-transcendent values are emphasized;
- Admiration enhances sustainable behavior when self-enhancement values are emphasized;
- Gratitude exerts a greater influence on sustainable behavior than admiration when self-transcendent values are prominent;
- Admiration exerts a greater influence on sustainable behavior than gratitude when self-enhancing values are prominent.

### 2.5 The mediating role of self-efficacy

The influence of various positive emotions associated with human values on sustainable behavior can be elucidated through the concept of self-efficacy (Yan et al., 2024). Utilizing the same theoretical framework, we hypothesized that the influence of gratitude and admiration’s alignment with self-transcendent and self-enhancement values on sustainable behavior can be elucidated through self-efficacy.

Self-efficacy refers to an individual’s confidence in their capability to execute a behavior necessary for achieving a particular task (Locke and Bandura, 1987). In consumer behavior, self-efficacy refers to the beliefs consumers hold regarding their ability to perform specific behaviors and the anticipated outcomes of those behaviors (Bandura et al., 1999). In this study, self-efficacy denotes consumers’ confidence in executing sustainable behaviors and their belief in the positive environmental impact of these actions.

Studies indicate that self-efficacy influences an individual’s effort and persistence in specific activities (Bandura, 1978). Individuals exhibiting high self-efficacy tend to engage in challenging tasks and demonstrate greater willingness to exert effort and persist longer when confronted with difficulties (Bandura et al., 1999; Bandura, 1978). Sustainable behavior necessitates a long-term commitment from individuals toward environmental protection and presents significant challenges. Numerous scholars have confirmed the influence of self-efficacy on predicting consumer attitudes and tendencies regarding sustainable behavior (White et al., 2011; Schutte and Bhullar, 2017). Schutte and Bhullar (2017) found that individuals with higher self-efficacy exhibit greater motivation to engage in sustainable behaviors. Further research indicate that when consumers’ emotional states correspond with value criteria, their confidence in evaluating product choices is enhanced (Adaval, 2001). We propose that the alignment of gratitude and admiration with self-transcendent and self-enhancement values may enhance sustainable behavior by improving perceived self-efficacy.

**Figure 1:**
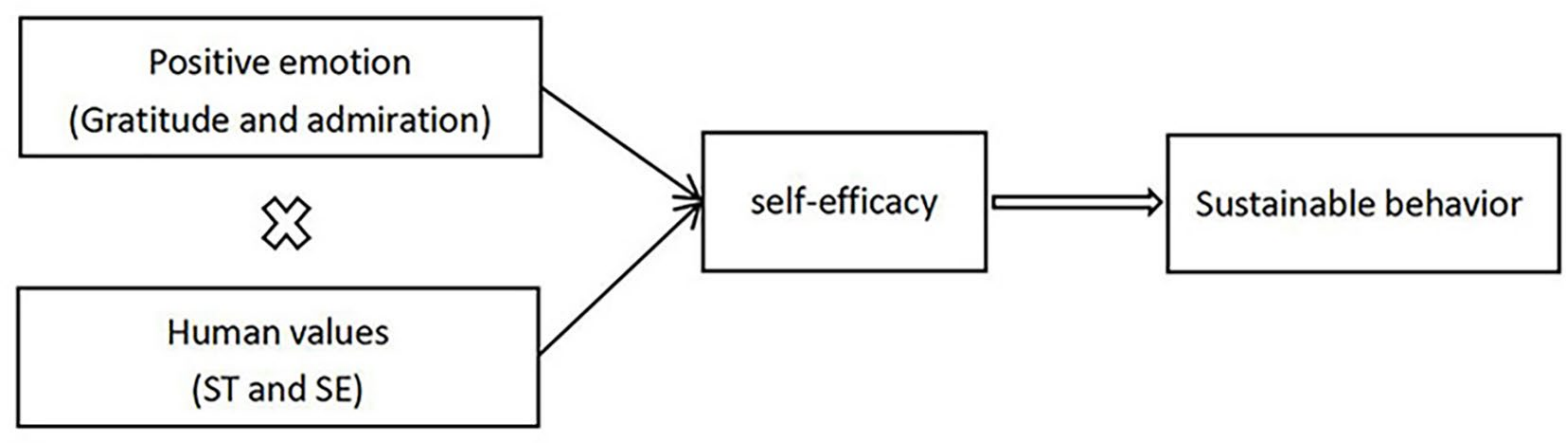
Research model.

Previous research indicate that gratitude correlates with self-transcendent value (Haidt, 2003), while admiration is linked to self-enhancing value. When individuals’ emotions align with their values, consumers experience a sense of “appropriateness” regarding their actions or intentions (Yan et al., 2024; Camacho et al., 2003; Kruglanski, 2006). Emotion-value fit is posited to enhance consumers’ self-efficacy in achieving specific goals (Kruglanski, 2006; Higgins, 2006). The alignment of emotions and values can increase consumer confidence and positively affect the implementation of sustainable behaviors. Studies have established that elevated self-efficacy enhances sustainable behaviors in consumers, including increased willingness to recycle and greater recycling activities (White et al., 2011; Schutte and Bhullar, 2017). We propose the following hypothesis:

*H2:* The alignment of various positive emotions (gratitude and admiration) with human values (self-transcendence and self-enhancement) enhances self-efficacy, thereby promoting sustainable behavior (Figure 1).

## 3 Experimental design and analysis

### 3.1 Initial testing

This pre-experiment aimed to evaluate the validity of the selected stimuli for sustainable behaviors in the study. Existing studies identify various stimuli for sustainable behaviors, with key examples including sustainability registration (such as plastic-free registration activities), recycling behaviors, and green purchasing behaviors (for instance, green purchasing intentions). This paper selects three representative stimuli from the existing literature: plastic-free registration activities, donation activities, and green purchasing intentions. The experiment does not disclose any brand or merchant information when presenting the manipulator images.

The pre-experiment procedure involved several steps: participants first read the attribute descriptions of sustainability behaviors, then examined three provided images, and subsequently rated each behavior on a 5-point scale, where lower scores indicated less sustainability. Finally, participants selected the stimulus representing the sustainable behavior they preferred. The findings indicated that the subjects agreed more with the sustainability of the green purchasing behavior (M_Green purchasing_ = 4.53, M_Donation Activity_ = 4.37, and M_Plastic Free Registration Behavior_ = 4.29). Based on this, the survey proceeded to examine the subjects’ overall inclination toward the three behaviors. The results indicated that the subjects’ inclination toward green purchasing was the strongest, with M_Green Purchase tendency_ = 4.39, M_Donation Activity tendency_ = 3.86, and M_Plastic-free registration tendency_ = 3.63. As a result, the green purchase behavior was chosen as the manipulator of sustainable behavior.

### 3.2 Experiment 1

Study 1 investigated the interactive effects of two emotions, gratitude and admiration, alongside values of self-enhancement and self-transcendence, on purchase intentions for green products, which are a significant aspect of sustainable behavior. This study examines the values inherent in brand positioning to improve the practical applicability of our findings.

#### 3.2.1 Methodological design and procedures

A total of 280 subjects were recruited via the Credamo platform. The procedure involved dividing the subjects into four groups: “gratitude + self-enhancement,” “gratitude + self-transcendence,” “admiration + self-enhancement,” and “admiration + self-transcendence.” In order to construct a fictional brand YQ and generate four advertisements for it, the experimental manipulation of Yan et al. (2024) was employed, with the exception of the values and sentiments conveyed being distinct. We utilized “gratitude for your choice” to convey gratitude and “admiration for your choice” to articulate admiration. The value of self-enhancement was activated by asserting that the new eco-friendly products “convey status, prestige, and refined fashion tastes” and “enhance your athletic experience to achieve a greater self.” The new products emphasize self-transcendence by expressing responsibility and care for the environment, encouraging individuals to transcend personal interests for the betterment of the planet. To improve the self-enhancement values Initiative, participants will examine the statement that “the YQ brand is shifting its focus to producing quality products” and characterize the new eco-friendly sneaker as a “unique and prestigious product” featuring a “beautiful design” and aimed “for the betterment of the planet” and “for an enhanced customer experience.” Participants will read that “the YQ brand has shifted its focus to making sustainable products” and describe the new eco-friendly sneakers as “available to everyone who wants to help protect the environment,” “with social welfare in mind,” and “for a better environment.” Before the formal experiment, participants rated seven emotions (gratitude, admiration, happiness, sadness, fear, disgust, and anger), assessed the values depicted in the posters based on their alignment with self-transcendence and self-enhancement values (1 = not at all, 7 = completely), and evaluated the effectiveness of the manipulation in relation to the rating data. Participants were randomly assigned to view one of four advertisements, after which they indicated their willingness to purchase the green sneakers.

#### 3.2.2 Results and discussion

**Figure 2:**
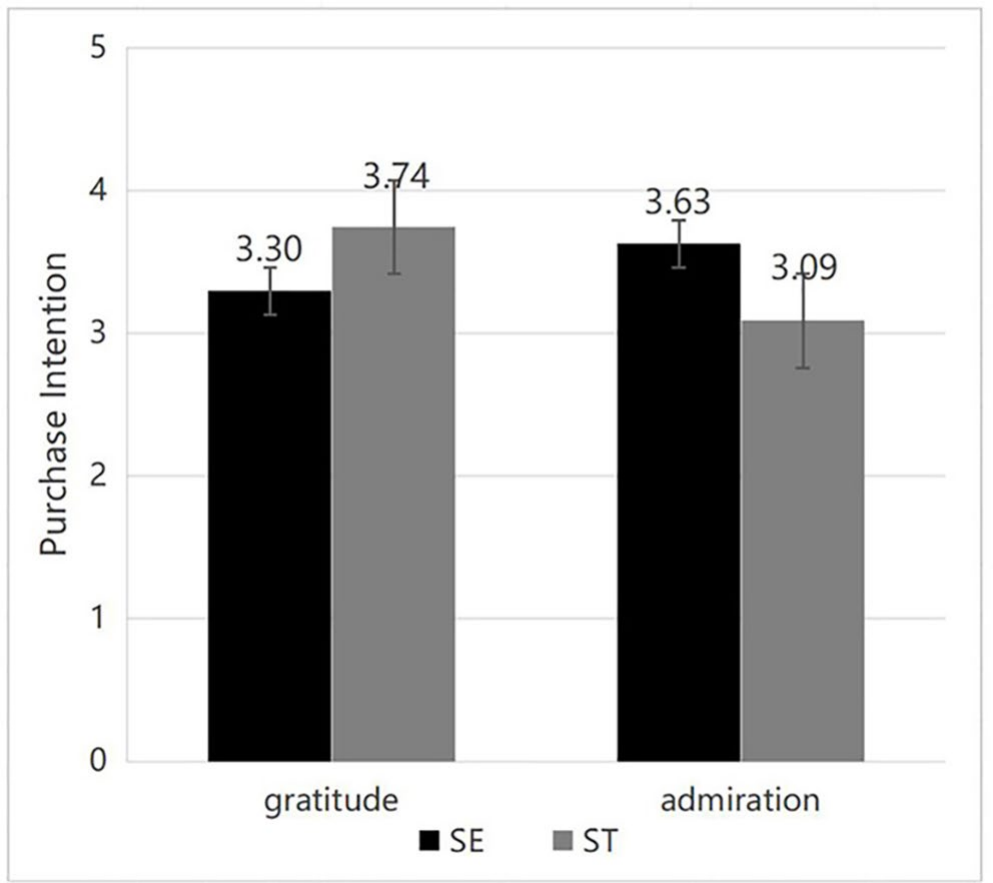
Purchase intention.

1. Demographic data of the participants. The subjects exhibit a more equitable distribution between men and women, predominantly comprising younger demographics, which represent the primary drivers of contemporary consumption. Over 60% of the subjects possess a bachelor’s degree or higher and demonstrate strong reading comprehension skills, enabling them to better grasp the experimental content. However, as they predominantly belong to a younger demographic, their income levels are relatively low (Table 1).
2. Reliability and validity assessment. The willingness-to-buy scale’s reliability test in Experiment 1 reveals that its Cronbach’s coefficient is 0.88 > 0.8, indicating that the scale is more reliable. The KMO value and Bartlett’s sphericity test guarantee the accuracy of the final measurement data. This purchase intention scale has a KMO value of 0.83, which is higher than 0.8, thus it has good measurement validity.
3. Emotional manipulation assessment. In the gratitude emotion manipulation test, participants in the gratitude condition reported a greater level of gratitude compared to those in the admiration condition, demonstrating the effectiveness of the gratitude manipulation. M_Gratitude (enhancement)_ = 3.59, SD = 1.99; M_Gratitude (transcendence)_ = 4.37, SD = 1.97. M_Admiration (self-enhancement)_ = 2.69, SD = 1.27. M_Admiration (self-transcendence)_ = 2.77, SD = 1.23; *F*(3, 276) = 16.00, *p* < 0.001. In a similar vein, participants in the admiration manipulation condition reported a greater level of admiration compared to those in the gratitude condition. M_Gratitude (enhancement)_ = 3.14, SD = 1.55; M_Gratitude (transcendence)_ = 3.53, SD = 1.47; M_Admiration (enhancement)_ = 4.43, SD = 1.90; M_Admiration (transcendence)_ = 5.37, SD = 1.48; *F*(3, 276) = 26.70, *p* < 0.001. The study indicated that other emotions related to the gratitude and admiration manipulation were not statistically significant, as presented in Table 2.
4. Values manipulation assessment. The experimental data indicated that subjects in the self-enhancement (SE) manipulation state reported higher levels of self-enhancement values compared to those in the self-transcendence (ST) state. M_SE (gratitude)_ = 4.86, SD = 1.45; M_SE (admiration)_ = 5.03, SD = 1.43; M_ST (gratitude)_ = 4.18, SD = 1.29; M_ST (admiration)_ = 3.64, SD = 1.68; *F*(3, 276) = 15.46, *p* < 0.001. Subjects in the self-transcendence (ST) manipulation condition reported higher levels of self-transcendent values compared to those in the self-enhancement (SE) condition. M_ST (gratitude)_ = 5.46, SD = 1.11; M_ST (admiration)_ = 5.59, SD = 1.19; M_SE (gratitude)_ = 4.26, SD = 1.53; M_SE (admiration)_ = 4.04, SD = 1.32; *F*(3, 276) = 26.79, *p* < 0.001. The experiment successfully manipulated self-enhancement and self-transcendence values.
5. Interaction effects. The interaction effect of emotions and values was examined, with emotions and values serving as independent variables and willingness to buy as the dependent variable. The results are presented in Table 3. The interaction effect between emotions and values was significant, *F*(1, 276) = 26.55, *p* < 0.001, *η*^2^ = 0.09. Subsequent analysis of simple effects indicated that gratitude significantly enhances green purchase intention when self-transcendent values are emphasized, as illustrated in Figure 2. Furthermore, the influence of gratitude on green purchase intention was found to be more pronounced than that of admiration emotions under conditions of significant self-transcendent values. M_Admiration_ = 3.09, *p* < 0.001, M_Gratitude_ = 3.74, and H1a and H1c hypotheses were confirmed. Admiration enhances green purchase intention more effectively when self-enhancement value is prioritized. Furthermore, when self-enhancement value is significant, the influence of admiration on green purchase intention surpasses that of gratitude sentiment. M_Gratitude_ = 3.30, M_Admiration_ = 3.63, *p* < 0.005, and H1b and H1d hypotheses are confirmed. Consequently, H1 was confirmed, indicating an interaction between discrete positive emotions and human values.

**Table 1:** Demographic information of individuals (Experiment 1).

Statistical variable	Classification	Counts	Frequency	Cumulative frequency
Gender	Man	147	52.50%	52.50%
	Female	133	47.50%	100.00%
Age	0–20	41	14.60%	69.70%
	21–30	231	82.50%	14.60%
	31–40	8	2.90%	97.10%
	40 and above	0	0.00	100.00%
Education	High school and below	5	1.80%	1.80%
	Vocational Secondary School	5	1.80%	3.60%
	Junior college	8	2.90%	6.40%
	Bachelor degree	178	63.60%	70.00%
	Master’s degree or above	84	30.00%	100.00%
Monthly income	2,000 and below	129	46.10%	46.10%
	2,000–4,000	95	33.90%	80.00%
	4,000–6,000	39	13.90%	93.90%
	6,000 and above	17	6.10%	100.00%

**Table 2:** Emotional manipulation test.

Emotion	Gratitude SE	Gratitude ST	Admiration SE	Admiration ST	*F*	*p*
Happy	2.91 (1.53)	3.37 (1.78)	3.21 (1.39)	3.21 (1.68)	*F* (3,276) = 1.11	0.036
Sad	1.53 (1.01)	1.93 (1.40)	1.99 (1.36)	1.70 (1.00)	*F* (3,276) = 2.14	0.096
Fear	1.41 (0.89)	1.83 (1.48)	1.70 (1.26)	1.51 (0.91)	*F* (3,276) = 1.78	0.151
Hate	1.46 (1.02)	1.79 (1.36)	1.83 (1.08)	1.39 (0.84)	*F* (3,276) = 2.29	0.031
Anger	1.44 (0.83)	1.59 (1.12)	1.70 (1.29)	1.30 (0.67)	*F* (3,276) = 2.59	0.053

**Table 3:** Cross-effect test.

Group	DOF	MS	*F*	*p*
Value	1	0.15	0.24	0.628
Emotion	1	1.89	2.95	0.087
Value × Emotion	1	17.00	26.55	0.000

### 3.3 Experiment 2

Experiment 2 aimed to examine the mediating effect of self-efficacy in the relationship between pleasant emotions (gratitude and admiration) and the sense of life worthiness (self-enhancement and self-transcendence) on the intention to purchase green items.

#### 3.3.1 Methodological design and procedures

To enhance the robustness of the experiment and re-recruit 280 individuals on the Credamo platform, we initially elicited the emotions of gratitude and admiration through the recall method. Participants were instructed to recall and detail their experiences of the target emotions (Septianto et al., 2021; Xie and Chun, 2019). To ensure a shared understanding among participants, we included a definition of the target emotion in the instructions. Upon completion of the writing task, participants will evaluate the intensity of emotions experienced, such as gratitude, admiration, and awe. Following the recall, participants were directed to a shopping scene featuring backpacks constructed from recycled materials. Participants were randomly assigned to view one of two advertisements. The ST (Self-Transcendence) advertisement employed the phrases “go beyond your own personal interests to create a better world,” “you can make a socially responsible purchase by purchasing this backpack,” and “promote the welfare of others.” The promotion of the green backpack emphasizes the wellbeing of others, while self-enhancement advertisements focus on personal benefits, using phrases like “enhance your own personal outcomes” and “express yourself through the backpack.” The SE (Self-Enhancement) advertisement for the Green Backpack employs phrases such as “enhance your own personal outcomes; you can achieve a better you by purchasing this backpack” and “express yourself through the backpack to convey your status, prestige, and exquisite taste.” Following exposure to an advertisement for the green backpack, participants assessed their intent to purchase the backpack (Yan et al., 2024).

#### 3.3.2 Results and discussion

**Figure 3:**
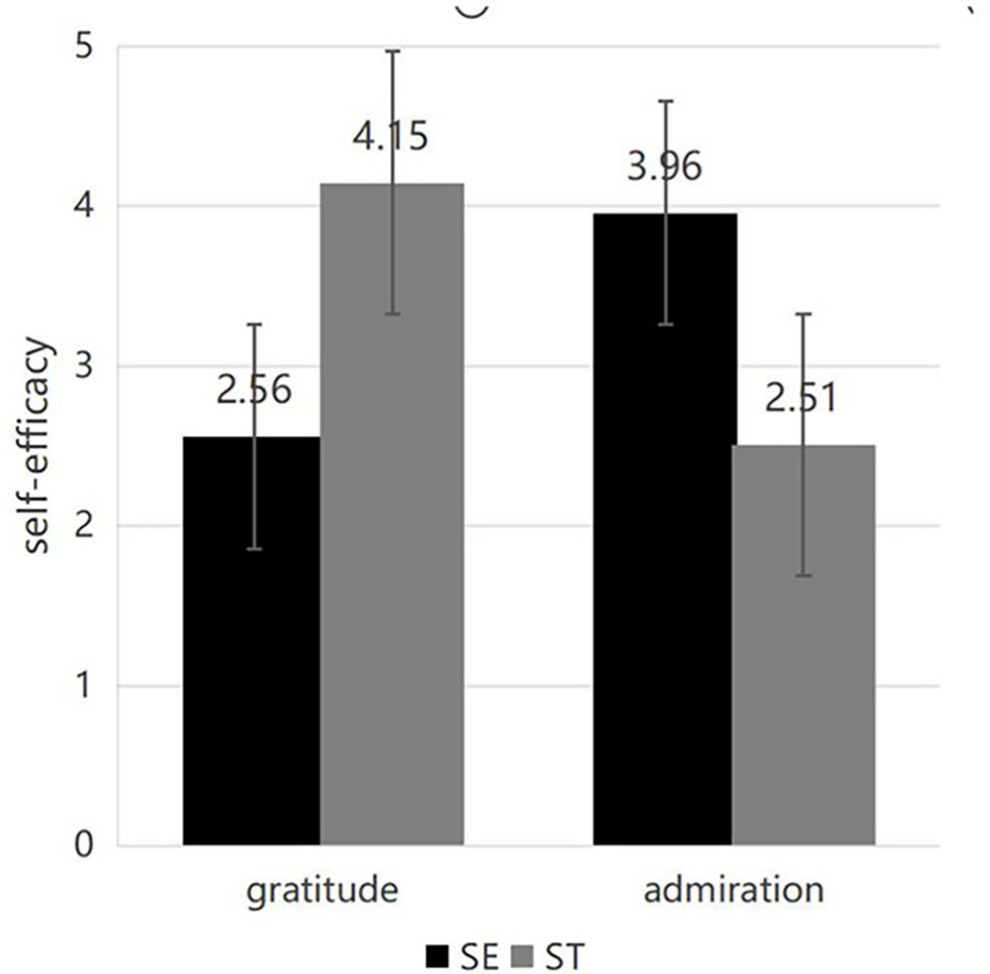
Self-efficacy.

**Figure 4:**
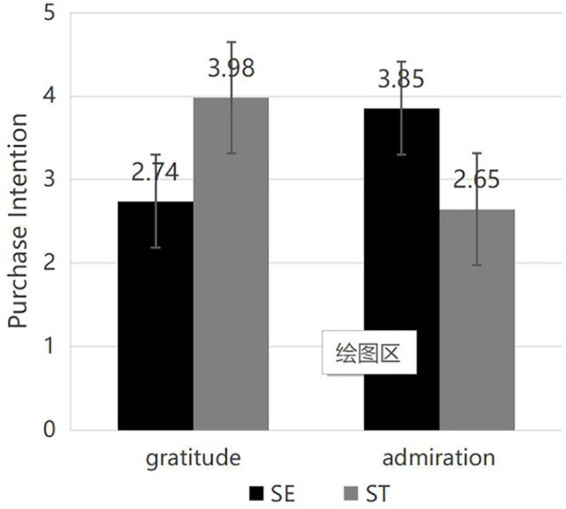
Purchase intention.

1. Demographic data of the participants. The subjects exhibit a more equitable distribution between men and women, predominantly comprising younger demographics, who represent the primary drivers of contemporary consumption. Over 70% of the participants possess a bachelor’s degree or higher, resulting in enhanced reading comprehension skills that facilitate a better understanding of the experimental content (Table 4).
2. Reliability and validity assessment. The self-efficacy and willingness to buy scales’ reliability tests in Experiment 2 reveal that the self-efficacy scale’s Cronbach’s coefficient is 0.91 > 0.8 and the willingness to buy scale’s Cronbach’s coefficient is 0.92 > 0.8. These results demonstrate the scales’ superior reliability, which is confirmed by the KMO value and the Bartlett sphericity test. Bartlett’s test of sphericity verifies the validity of the final measurement outcomes. The self-efficacy scale’s KMO value is 0.85, and the desire to buy scale’s KMO value is 0.85 as well. These values show that the scale has good measurement validity.
3. Emotional manipulation assessment. In the gratitude emotion manipulation test, subjects exposed to the gratitude manipulation reported a higher level of gratitude compared to those in the admiration manipulation state, demonstrating the effectiveness of the gratitude emotion manipulation. M_Admiration_ = 3.72, SD = 1.50; M_Gratitude_ = 5.84, SD = 1.37; *F*(1, 278) = 151.72, *p* < 0.001. In the admiration emotion manipulation test, subjects experiencing the admiration state reported a higher level of admiration compared to those in the gratitude state, demonstrating effective manipulation of the admiration emotion. M_Gratitude_ = 4.38, SD = 1.78; M_Admiration_ = 5.55, SD = 1.55, *F*(1, 278) = 42.15, *p* < 0.001. All remaining emotions, with the exception of the positive discrete emotion of happiness, did not demonstrate statistical significance regarding negative emotions, as indicated in Table 5. The values manipulation test indicated that subjects in the self-enhancement (SE) manipulation state reported higher levels of self-enhancement values compared to those in the self-transcendence (ST) state, M_SE_ = 4.79, SD = 1.33; M_ST_ = 3.85, SD = 1.39; *F*(1, 278) = 33.63, *p* < 0.001. Subjects in the self-transcendence (ST) manipulation exhibited higher levels of self-transcendent values compared to those in the self-enhancement (SE) state, M_SE_ = 4.27, SD = 1.38; M_ST_ = 5.38, SD = 1.22; *F*(1, 278) = 50.67, *p* < 0.001, indicating the effectiveness of the values manipulation.
4. Mediating effects. The mediation effect test of self-efficacy was conducted using the Process plug-in in SPSS, with Hayes’ Model 8 as the moderated mediation model. The index of moderated mediation was examined to test if the model (Model 8) was significant. The confidence interval for the index did not contain zero (Index = −1.84, 95% CI [−2.21, −1.51]), suggesting the overall model was significant. The results are presented in the table. The mediation effect of self-efficacy is significant, with a value of 0.18 and a 95% confidence interval of [0.14, 0.23], which does not contain 0. This suggests that self-efficacy plays a mediating role in the process of the role of positive emotions and values on the willingness to buy green. The interaction term of positive emotions and values on self-efficacy is also significant in the path of the role of self-efficacy, with a 95% confidence interval of [0.20, 0.36]. The path of self-efficacy on green purchase intention was also significant, with a 95% confidence interval of [0.58, 0.72] that does not contain 0. The path of the interaction term of positive discrete emotions and values on green purchase intention was also significant, with a 95% confidence interval of [0.03, 0.13] (Table 6). In summary, self-efficacy served as a mediator in the interaction between positive discrete emotions and values, while values partially mediated the relationship between values and green purchase intention, as illustrated in Figures 3, 4. Self-efficacy was elevated when gratitude emotions aligned with self-transcendence values and admiration emotions aligned with self-enhancement values, resulting in increased green purchase intention, thereby validating Hypothesis H2.

**Table 4:** Demographic information of individuals (Experiment 2).

Statistical variable	Classification	Counts	Frequency	Cumulative frequency
Gender	Man	126	45.00%	45.00%
	Female	154	55.00%	100.0%
Age	0–20	40	14.30%	69.70%
	21–30	224	80.00%	14.30%
	31–40	15	5.40%	99.60%
	40 and above	1	0.40%	100.00%
Education	High school and below	0	0.00	1.80%
	Vocational Secondary School	4	1.40%	1.40%
	Junior college	16	5.70%	7.10%
	Bachelor degree	200	71.40%	78.60%
	Master’s degree or above	60	21.40%	100.00%
Monthly income	2,000 and below	104	37.10%	37.10%
	2,000–4,000	92	32.90%	70.00%
	4,000–6,000	48	17.10%	87.10%
	6,000 and above	36	12.90%	100.00%

**Table 5:** Emotional manipulation test.

Emotion	Gratitude	Admiration	*F*	*p*
Happy	4.68 (1.36)	4.19 (1.54)	*F* (1,278) = 8.04	0.005
Sad	2.22 (1.56)	3.31 (1.41)	*F* (1,278) = 0.23	0.629
Fear	1.81 (1.30)	1.82 (1.26)	*F* (1,278) = 0.01	0.926
Hate	1.45 (1.01)	1.47 (1.04)	*F* (1,278) = 0.03	0.861
Anger	1.48 (1.10)	1.56 (0.98)	*F* (1,278) = 0.47	0.492

**Table 6:** Intermediate effect test.

Mediating	Effect pathway	Effect	Boot SE	Boot LLCI	Boot ULCI
Indirect	Emotion × value → self-efficacy (a)		0.04	0.20	0.36
	Self-efficacy → purchase intention (b)		0.03	0.58	0.72
	Indirect effect	0.18	0.02	0.14	0.23
Direct	Emotion × value → purchase intention (c)	0.08	0.03	0.03	0.13

## 4 Research conclusions and insights

### 4.1 Conclusion of the study

The present research investigates the interaction effects of discrete positive emotions and human values on the relationship between emotions and cognition. Both studies indicated that sustainable behaviors may be promoted by the alignment of self-enhancement with admiration emotions, as well as the alignment of self-transcendence with gratitude emotions. This paper further validates the effectiveness of identifying human values, specifically self-enhancement and self-transcendence, as distinct positive emotions that promote green purchase intention and sustainable behavior (Yan et al., 2024). Self-efficacy was introduced as a mediating variable to demonstrate that positive discrete emotions tend to enhance self-efficacy when aligned with life values, which subsequently promotes sustainable behaviors more effectively. This paper examines two positive discrete emotions, gratitude and admiration, and their respective impacts on self-efficacy. The findings of this study may assist managers in leveraging these emotions to enhance the effectiveness of sustainability initiatives, including the promotion of green purchasing intentions.

This paper expands the research on positive emotions by examining the interaction effects of gratitude and admiration. It elucidates the mechanism through which these emotions interact with life values, specifically self-enhancement and self-transcendence, influencing green purchase intentions, with self-efficacy serving as a partial mediator. Previous studies have primarily examined the effects of positive emotions such as pride, awe, and happiness on sustainable behaviors (Su and Guo, 2024). In contrast, the emotions of gratitude and admiration have received less attention. This study further elucidates the interaction effect of these positive emotions (gratitude and admiration) and human values (self-enhancement and self-transcendence) on green purchase intention, particularly from the perspective of self-efficacy. Existing studies predominantly examine external factors, including products, advertisements, brand image, and corporate claims, while giving insufficient attention to consumers’ psychological emotions. This research enhances the theoretical framework of sustainable behavior, specifically regarding green purchase intention, from a psychological standpoint.

### 4.2 Implications for practice

Initial findings indicate that self-transcendent and self-enhancing emotions are linked to distinct value systems. Some researchers propose that self-transcendent emotions attract greater attention from others, while self-enhancing emotions tend to promote self-focus (Stellar et al., 2017). Further research indicates a strong correlation between self-transcendent emotions and self-transcendent values, as well as between self-enhancing emotions and self-enhancing values (Jacobs and McConnell, 2022). This indicates a significant relationship between emotions and values, which may also enhance pro-social behavior to a certain degree. This study selected gratitude and admiration to examine their relationships with self-enhancement and self-transcendence, respectively, and to investigate the effects of both on pro-social behavior. The present research indicates that gratitude is more likely to foster a sense of self-efficacy conducive to sustainable behavior when associated with self-transcendence values. Conversely, admiration is more likely to enhance self-efficacy related to sustainable behaviors when connected to self-enhancement values. These findings not only bear significance for environmental action but also have wider implications for both pro-social and self-serving behaviors. The concept of change occurring alongside emotions and values aligns with the social intuitionist model (Haidt, 2003), which emphasizes the role of emotions in driving the development of beliefs and values. Future research may investigate the extent to which emotions and values facilitate pro-social and pro-environmental behaviors as suggested by this theory.

Marketers in the field of marketing should leverage the beneficial impacts of positive emotions and life values to encourage specific sustainable behaviors, including sustainable consumption behaviors (Yan et al., 2024). This investigation employs two analyses to demonstrate how companies can leverage various positive emotions to enhance the effectiveness of their sustainable behavior campaigns. The emotion of gratitude is employed in marketing to align with the value of self-transcendence, which is instilled in consumers through advertisements and other mediums to enhance their purchasing intent. Organizations or brands must effectively operationalize values that align with consumers’ spirituality and are conducive to eliciting positive emotions. This includes utilizing images or audio that correspond to values like self-enhancement or self-transcendence in environmentally friendly advertising campaigns to evoke emotions such as gratitude or admiration. Our findings offer pertinent recommendations and illustrations of how companies can effectively leverage discrete positive emotions and life values to attain desired marketing outcomes, including the enhancement of consumers’ purchase intentions. This research aids corporate managers in identifying positive discrete emotions that align with values such as self-enhancement, thereby promoting sustainable consumption behaviors among their target demographics through an effective combination.

With the emergence of sustainable consumption and green marketing, companies have increasingly engaged in “greenwashing” practices to enhance profitability. This involves communicating to consumers the behavioral advantages of the company’s environmental initiatives or the ecological impacts of its products or services (Baum, 2012). While it enhances shareholder benefits to some degree, it significantly diminishes overall societal benefits when viewed through the lens of resource allocation and social welfare (Yang et al., 2020). Consequently, it is essential to implement specific measures to minimize such behavior. The government should clearly define and categorize “greenwashing” within relevant laws and regulations, impose penalties for such behavior, and safeguard legitimate green enterprises (De Vieira et al., 2020). Green businesses can be defined and categorized within an organizational framework. At the organizational level, green companies may implement a third-party certification platform to oversee the ethical standards of green marketing; however, it is essential to ensure the fairness of this certification through specific measures. Society should enhance consumers’ capacity to identify green products by encouraging recognition of green product logos and implementing additional measures to increase consumer autonomy (Szabo and Webster, 2021).

### 4.3 Constraints and future directions

This paper examines the new categorical dimension of positive discrete emotions to investigate the influence of interaction with values on sustainable behaviors. It analyzes the internal mechanisms involved and explores the mediating role of self-efficacy from a consumer psychology perspective during this interaction, while acknowledging certain limitations. The study primarily examines young individuals, specifically college students. The premium nature of green consumption, coupled with the limited income of young individuals, necessitates an enhancement of the study’s external validity through an expanded research scope. Secondly, regarding the manipulation of experimental scenarios, the primary methods employed include text and images to present product advertisements. Future research could incorporate audio and other modalities to generate a more vivid representation of the advertising stimulus effect, thereby creating a more authentic advertising impact and improving the overall effectiveness of the experiment. Moreover, contemporary studies on values predominantly emphasize self-transcendence values rather than self-enhancement values. Future investigations should explore the evaluation criteria of additional value dimensions (Yan et al., 2024). This paper focuses on two positive emotions, gratitude and admiration. Future research may investigate additional positive emotions, including happiness and wellbeing. Furthermore, consumers experience various negative emotions, such as aversion and fear. It is important to examine whether these negative emotions align with relevant values and how they may influence consumers’ sustainable behavior.

## Data Availability

The original contributions presented in the study are included in the article/supplementary material, further inquiries can be directed to the corresponding author.
